# Influence of Ultrasound Examination on Diagnosis and Treatment of Temporomandibular Disorders

**DOI:** 10.3390/jcm11051202

**Published:** 2022-02-23

**Authors:** Małgorzata Pihut, Andrzej Gala, Rafał Obuchowicz, Karolina Chmura

**Affiliations:** 1 Prosthodontic Department, Dental Institute, Jagiellonian University Medical College, 30-663 Krakow, Poland; malgorzata.pihut@uj.edu.pl (M.P.); andrzej.gala@uj.edu.pl (A.G.); 2 Department of Diagnostic Imaging, Jagiellonian University Medical College, 30-663 Krakow, Poland; r.obuchowicz@gmail.com

**Keywords:** temporomandibular disorders, ultrasound, therapy

## Abstract

Background: Disorders of the masticatory muscles and temporomandibular joints as well as the surrounding craniofacial structures are called temporomandibular disorders. These are dental diseases affecting an increasing number of people with a multifactorial etiology. Noninvasive ultrasonography imaging of temporomandibular joints was performed to obtain more detailed information on joint pathologies. Material and Methods: The aim of the study was to assess the influence of ultrasound examinations of the temporomandibular joints on the diagnosis and treatment planning in patients with temporomandibular disorders. The study included 110 patients examined with the use of the Research Diagnostic Criteria for Temporomandibular Disorders questionnaire, axis I and II, after which the initial treatment plan was created. All patients underwent an ultrasound examination of the temporomandibular joints. Results: The results reveal numerous morphological changes within the joint structures in all treatment groups. Comparative statistical analyses of symptoms were performed between study groups. Conclusions: The number of pathologies in the myofascial pain group was much higher than expected and required introduction of additional treatment procedures. Further studies confirming these results and the effectiveness of ultrasound diagnostic of temporomandibular disorders are recommended.

## 1. Introduction

Heretofore, temporomandibular disorders (TMD) have been primarily perceived as functional disorders occurring in the masticatory system that are not accompanied by morphological changes. The use of advanced visualization technics, for instance, the MRI, has proved that TMDs also include pathological anatomical alterations of temporomandibular joints, as well as the surrounding craniofacial structures and masticatory muscles [1,2,3,4,5]. It is a common dental disease affecting an increasing number of people in the population. TMDs’ etiology is multifactorial. The main symptoms are masticatory muscle pain and/or temporomandibular joint (TMJ) pain, and a clicking sound in joints, resulting from the pathological displacement of the articular disc with reduction or limitation of the jaw opening range [3,4,6,7].

The main goal of TMD management is pain elimination in the masticatory muscles, often caused by the long-term occurrence of occlusal parafunctions and excessively high muscle tension as well as restoration of symmetrical movement and minimalization of pathomorphological changes in TMJs’ intra-articular structures [1,3,5].

The complexity of TMDs might require using additional imaging techniques to obtain proper diagnosis and plan effective treatment. The following techniques can be distinguished: panoramic radiography, transcranial radiographical projections of TMJs (with different angulations), computed tomography (CT), cone beam computed tomography (CBCT), magnetic resonance imaging (MRI) and ultrasonography (USG). Flat plane, conventional radiography is highly available in dental offices. However, it has limited utility in TMJ examination as it only shows advanced changes in hard tissue structures. In CBCT and CT, only bone structures are assessed. Lesions such as bone erosions, fractures and deformations can be detected. CT scans involve high X-ray exposure and are not suitable for soft tissue assessment. MRI is considered the gold standard for the evaluation of soft tissues and the method of choice for imaging articular discs. Moreover, the use of an appropriate sequence enables the analysis of the TMJs’ mobility range. It can detect degenerative changes at an early stage. However, its main disadvantages, such as the high cost, limited availability, long examination time and exposure to magnetic fields, exclude its use in some patients and require the application of alternative imaging techniques [1,3,8].

Medical ultrasound is a noninvasive application of high-frequency sound waves for the examination and imaging of soft tissues. Studies have already shown that it can be used in dentistry as an imaging modality to analyze the temporomandibular joint in cases of temporomandibular disorders [8,9,10,11]. It enables changes detection in small organs (from 0.1 mm) with high accuracy. It can be performed on pregnant women and children and can be repeated many times. Moreover, it allows a dynamic examination of the temporomandibular joints, which means that structures are also visible during the movements of the jaw [12,13,14,15,16]. According to several references [8,9,14,15] and personal experience, ultrasound examinations (USG) often reveal that functional pathologies are accompanied by numerous pathomorphological changes in the temporomandibular joints. It might be an indication for the use of additional therapeutic methods as support for those routinely used. 

The indications for ultrasound examination include injuries and inflammation of joints, tendons, ligaments, soft tissues and bones, especially in post-traumatic conditions or pain syndromes and limited mobility in joints. Furthermore, ultrasound examination is useful in the diagnosis of degenerative diseases or cases of interventional procedures (intra-articular injections, punctures, biopsies). The studies described below have also shown that monitoring treatment progress is significantly useful [14,15,17,18,19,20]. The aim of the study was to assess the TMJ ultrasound results and their influence on diagnosis and treatment planning in TMD patients.

## 2. Materials and Methods

The study population was recruited from patients referred to the Consulting Room of Temporomandibular Disorders, Institute of Dentistry Medical College due to the presence of a painful form of TMD. Research inclusion criteria were: presence of TMD, the appropriate age range (18–50 years) and no contraindications for appliance of ultrasound examination (open wounds, skin burns or bone injury over examination area). All patients had full dentition or missing teeth replaced with fixed dentures. The exclusion criteria were the occurrence of general diseases that would have made it impossible to continue participation or the patient’s willingness to withdraw.

Patients included in the study underwent: basic dental examination and diagnostics of temporomandibular disorders using the RDC/TMD questionnaire, axis I and II. Additional examinations consisted of a panoramic radiograph and ultrasound examination of the TMJs.

**Scheme 1:**
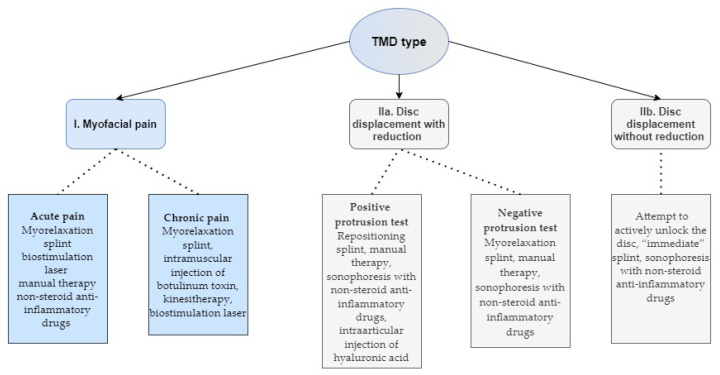
Types of temporomandibular disorders and methods of treatment.

After the first diagnostic test, the patient’s treatment was planned with the use of methods presented in the Scheme 1. These are standard and routinely performed initial treatments for patients based on literature and personal experience (plan I) [1,2,3,5,7,8,21]. Treatment introduction was postponed until the patient underwent USG examination.

The examination was performed and interpreted by (R.O.—co-author), who has over 20 years of clinical experience, and who was blinded to the clinical history of patients. It was conducted with the Philips IU22 and the 9–16 MHz hockey-stick-head type. During the test, it was arranged linearly, parallel to the course of the zygomatic arch. USG was introduced in the second stage to ensure the initial diagnosis’s correctness and further application of the most effective treatment plan. All patients gave written consent to the proposed diagnostic and therapeutic procedures. Main changes looked for in USG images were: effusion, thickness of the condylar disc, synovial thickness, condylar erosion, degenerative and overload changes of joint surfaces and features of ligament apparatus failure. 

Statistical analyses were performed in the STATISTICA (Tibco) v.13.3. It included descriptive statistics, as in the scheme presented below. Shapiro–Wilk test was calculated to check distributional adequacy. Statistical differences between group I and II were calculated by the Mann–Whitney test. To assess the differences between groups in terms of TMD, frequency and independence analyses using chi-square tests were performed. A *p*-value of less than 0.05 was deemed as statistically significant. The research was approved by the University Bioethics Committee 1072.6120.138.2017. All the patients gave their written consent to participate in the research project.

## 3. Results

The study population consisted of 110 patients, aged 21 to 44, both sexes (80 female, 30 male). According to the RDC/TMD diagnostic procedure, axis I, the following cases of temporomandibular disorders were identified. In the group of 68 (61.8%) patients, the myofascial pain form of dysfunction (form I) was diagnosed. The initial treatment plan included application of myorelaxation splints, and in the case of the chronic pain, additional intermuscular injections of botulinum toxin type A were given (cases with hypertonic masticatory muscles and lack of contraindication). In a few cases, nonsteroid anti-inflammatory drugs were administered. Physiotherapeutic procedures were carried out as follows: biostimulation laser, manual therapy and kinesiotherapy. A total of 24 patients were diagnosed with acute pain and 44 with chronic pain. A total of 30 patients (27.3%) were classified into group II a (according to Scheme 1) because of a disc displacement with reduction diagnosis. A total of 17 patients (56.7%) had positive protrusion tests and 13 (43.3%) had negative tests. The first plan included application of the repositioning splints, intraarticular injection of hyaluronic acid (positive protrusion test) or a myorelaxation splint (negative protrusion test). Additionally, manual therapy and sonophoresis with non-steroid anti-inflammatory drugs were administered. Disc displacement without reduction (II b) was diagnosed in the group of 11 patients (10% of the study population). At first, an attempt to actively unlock the disc was made. Then, an “immediate” splint of hard impression silicone material was used. Since at an early stage there is no reduction in the articular disc in a case of anterior disc replacement, to achieve the reposition of the articular disc, the initial proposal of an immediate splint such as described above is needed. Planned and conducted therapeutic methods were compliant with the current TMD treatment principles and the guidelines included in the literature (Scheme 1) [1,2,3,5,7,12,20,21,22,23,24].

### Ultrasound Examinations Results

**Figure 1:**
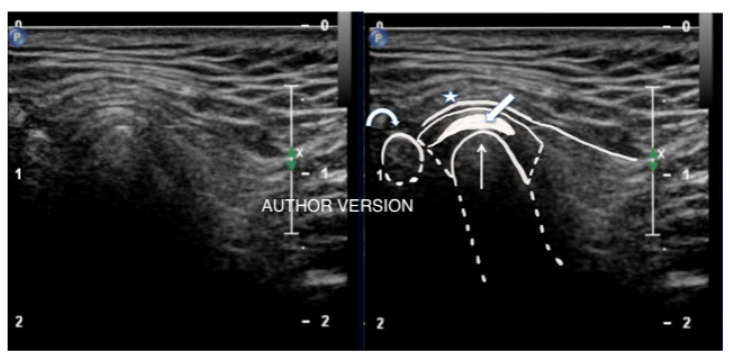
View of a healthy temporomandibular joint without pathological lesions with anatomical structures outlined.

The results of the ultrasound examinations revealed numerous morphological changes in the temporomandibular joints of 102 patients (92.7%), regardless of TMD duration. Examination results of eight patients (7.3%) were within the normal range (Figure 1).

**Scheme 2:**
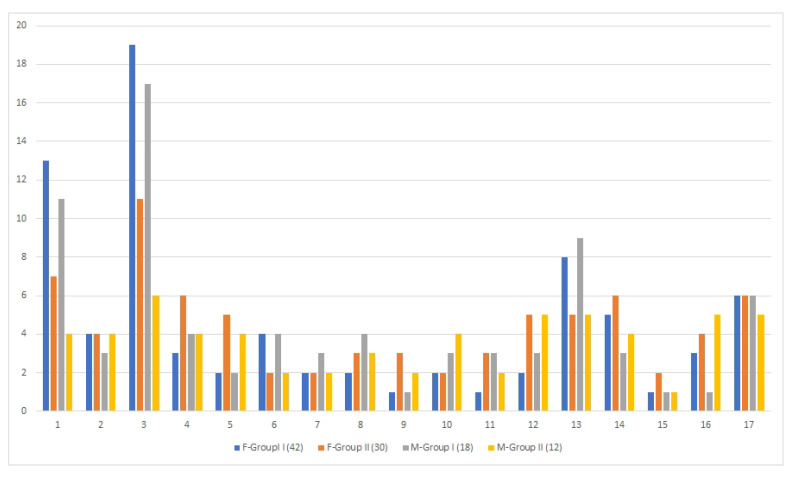
Distribution of TMD symptoms between group I and II for females (F) and males (M).

**Scheme 3:**
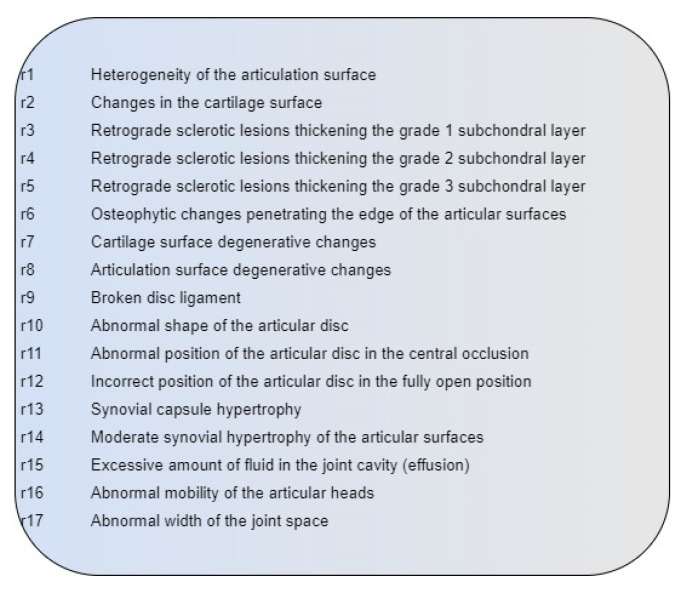
Changes revealed in the ultrasound examination.

For statistical analysis, patients were divided into two groups: group I and group II (according to RDC/TMD). Descriptive statistics of pathologies revealed in ultrasound examination were collected in Scheme 2 and Scheme 3.

A total of 70% of patients (42) in the myofascial pain group (I) were female. Statistical comparisons were made between groups I and II. The analysis did not show any significant differences between the groups with regard to gender: Z = −0.268; *p* = 0.789. The mean rank in group I was M = 52.00, while in group II it was M = 53.30. The analysis between group I and II also showed no significant differences between them: Z = −0.966; *p* = 0.334. The mean rank in group I was M = 49.17, while in group II it was M = 54.83.

**Figure 2:**
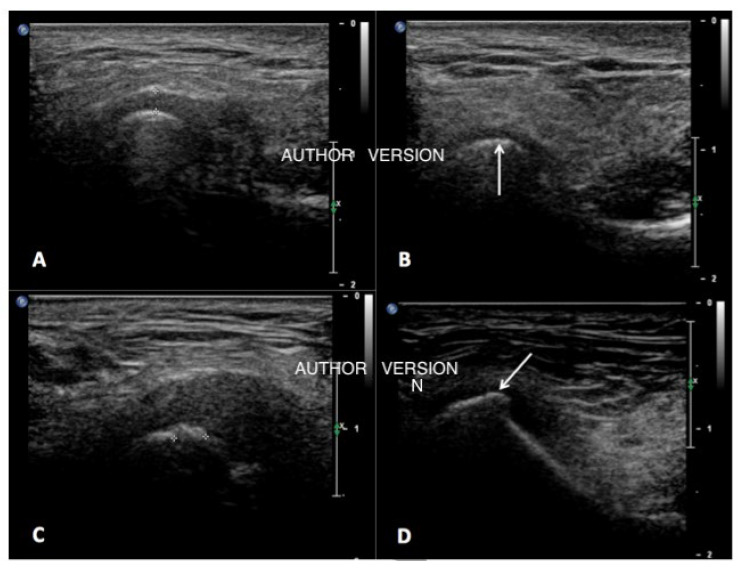
Different stages of condylar head articular surface degeneration visible on ultrasound pictures. Normal smooth surface of the condylar head (**A**). Subchondral sclerotic changes-typical for overload (arrow) (**B**). Cartilage destruction with penetration of productive changes (marked by clippers) (**C**). Osteophyte formation (arrow) (**D**).

**Figure 3:**
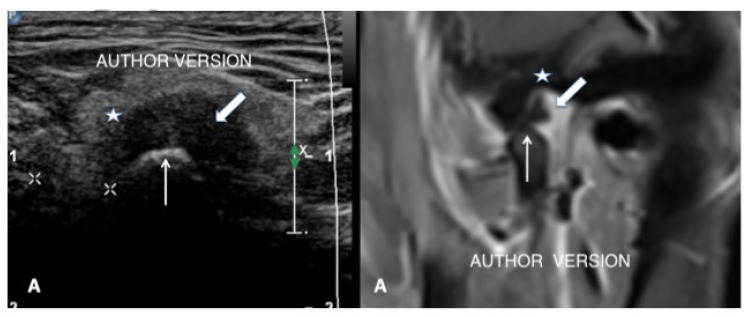
View of exudate causing increased width of the temporomandibular joint gap on ultrasound examination and MRI (star-glenoid fossa, thick arrow-disc, thin arrow-mandible condylar head.

The above results, when compared with the first treatment plans and pathomorphological changes occurring in the temporomandibular joints (Scheme 2), exposed that the initial treatment plan was insufficient. Additional methods were added to achieve optimal treatment procedures. In most cases, implementation of intra-articular injections of hyaluronic acid or platelet-rich plasma was necessary. Both substances exhibit significant healing properties for damaged soft structures of the joints (articular surfaces, disc, posterior disc ligament). They also increase the amount of internal fluid. The indications for their use were: degenerative changes, overload in the articular surfaces, features of ligamentous apparatus insufficiency, productive changes in the nature of layers, bone faults in the articular surfaces and osteophytic changes. Examples of different revealed pathologies are presented in Figure 2 and Figure 3.

## 4. Discussion

An interesting finding of this study is that the statistical comparison between two groups (muscle form-I and disc displacement-II) did not show any expected differences. Only eight of the examined patients did not exhibit pathological changes in the ultrasound examination of TMJ. Moreover, 88.2% of patients from the muscle group I showed similar TMJ changes to those in group II. TMD diagnostics and choosing the right therapeutic methods, even with the use of evidence-based questionaries such as RDC/TMD, are still a complex issue. Therefore, a significant amount of research is aimed at improving it. The prevalence of TMD is systematically increasing. Kundu H and colleagues [11] showed that about 6% to 12% of the population experience clinical symptoms of TMD. Patients present a broad age range, with a peak occurrence between 20 and 40 years of age; TMD has also been proven to be more common in women than in men. In turn, a few projects have been carried out assessing the usefulness of USG examinations [13,14,15,16,17].

This study suggests that ultrasound examination might be useful in revealing the development of TMJ changes in cases of patients who have not exhibited joint symptoms in the standard diagnostic process. However, this research has some limitations. Further studies on bigger sample sizes are necessary. Moreover, more even groups (both gender and type of disorder) are required to support its findings and verify if ultrasound imaging is an effective supportive method for the diagnosis of temporomandibular dysfunctions.

Klatkiewicz et al. [8] conducted a meta-analysis on ultrasound examinations used in the diagnosis of TMD and emphasized that ultrasound examination is a short-term, noninvasive, low-cost procedure for the patient and requires a short implementation time as well as high availability in healthcare facilities. However, results’ precision largely depends on the experience and skills of the doctors carrying out the examination and describing its results, which causes difficulties in its standardization [12,13,14,15,16].

Kalyan et al. [16] emphasized that existing equipment to assess the movement of the joints’ condyles (axiography, kinesiography, photocells and light-emitting diodes) are not sufficient to obtain satisfactory results. Therefore, the authors conclude that ultrasound examination is a valuable supplement to clinical tests and a valuable method of diagnosing intra-articular topography and joint damage [18,19,20,21]. TMJs are unique in many ways. Both joints move simultaneously, and their functional surface is made of fibrous connective [8]. In general, overview changes observed in this study included effusions, different stages of articular surface destruction, posterior disc ligament fibre damage, articular disc deformation and displacement. Numerous authors emphasize [9,14,17,19,22,23,24,25,26,27,28,29] that ultrasound examination is extremely accurate in determining the direction of the intra-articular disc displacement or it can rule it out. Furthermore, it provides valuable information for planning for an occlusal splint and choosing supportive treatment. In this study, the biggest number of supplementary procedures was found in the myofascial pain group (I). The list of observed pathologies was much longer than expected. Based on the literature, the results of USG offer the possibility of differential diagnosis in cases of inflammation (e.g., in juvenile rheumatoid or idiopathic arthritis) or the presence of degenerative changes, which are an indication for a different form of rehabilitation [27,28].

Carlos Fernando de Mello Junior et al. [23] compared ultrasound examination to magnetic resonance imaging. The author concluded that ultrasound examinations of the joints offer high sensitivity and specificity of results necessary for the proper diagnosis of TMD. Additionally, a study conducted by Janek et al. [9] showed high sensitivity (90%) and specificity (84%) in the assessment of the position of the articular disc in the central occlusion, as well as 94% sensitivity, 100% specificity and 94% accuracy in the case of degenerative changes. Alternatively, Manfredini et al. [29] reported a high number of false-negative and some false-positive results, suggesting USG is more specific than sensitive. Subsequently, a recent meta-analysis [8] showed much lower results for disc displacement, assessing its sensitivity as 76% and specificity as 69%. Differences in results and their variability still require better standardization techniques.

High-resolution USG, both in static and dynamic positions, is the preferred method for accurate results. It gives better diagnostic values than the static position alone. However, this procedure has a limited ability to detect medial disc displacement and visualize suspected perforations and adhesions visible on MRI. MRI should still be considered a gold standard in TMD diagnostics. Since ultrasound examination has unconditional pros, such as higher availability in a dental office, a lower cost, shorter time and nonionizing procedure, it should be researched as a useful supporting method for detection of temporomandibular joint abnormalities and planning noninvasive treatment [25,26,27,28,29,30,31].

## 5. Conclusions

1. Ultrasound results collected in this study showed a higher prevalence of pathomorphological TMJ changes in the myofascial pain group than expected based on clinical examination.
2. Further studies based on larger sample size are recommended to compare and verify if ultrasound imaging can be an effective new method supporting diagnoses of temporomandibular disorders.

## Data Availability

The data presented in this study are available on request from the corresponding author in the national language.
